# Interaction of FXTAS Family History and College Degree Attainment Predicts Trajectories of Cognitive and Motor Symptoms in *FMR1* Premutation Carrier Women

**DOI:** 10.1002/ajmg.b.70022

**Published:** 2026-05-21

**Authors:** Jinkuk Hong, Leann Smith DaWalt, Jessica Klusek, Elizabeth M. Berry-Kravis, Marsha Mailick

**Affiliations:** 1 Waisman Center, University of Wisconsin-Madison, Madison, Wisconsin, USA; 2 University of South Carolina, Columbia, South Carolina, USA; 3 Rush University Medical Center, Chicago, Illinois, USA

**Keywords:** college degree attainment, *FMR1* premutation carriers, genetic familial risk, neurodegenerative symptoms, neuroprotection

## Abstract

The present longitudinal study focuses on *FMR1* premutation carrier women during midlife and early old age (*n* = 115). Bringing together the genetic risk factor of a family history of FXTAS and the environmental protective factor of higher education, the goal of the study was to determine how these factors potentially interact to predict self-reported FXTAS-type symptoms in women carrying the *FMR1* premutation. We investigated age-related changes in symptoms, measured prospectively over a decade. Using an accelerated longitudinal design approach, we estimated trajectories of change extending from early midlife (starting at age 39) into older age (up to age 80) in a community-based cohort. Results indicated that women with the *FMR1* premutation who have a family history of FXTAS are at greater risk for experiencing symptoms during late midlife and older age than those who do not have such a family history. Consistent with research on other neurodegenerative diseases, this family history risk may be moderated by completing a college education, suggesting that cognitive enrichment during a period of neuroplasticity might provide protection from the neurodegenerative symptoms that are experienced by some women with the premutation as they age.

## 1 | Introduction

The present study employed a gene × environment framework to investigate how higher education attainment may be protective for individuals who have a family history of a specific type of neurodegeneration that is due to a variant in the *FMR1* gene. The *FMR1* gene (*fragile X messenger ribonucleoprotein 1*) plays a critical role in normal neurological development and functioning and regulates the translation of approximately 30% of all transcripts in the synaptic proteome (Darnell et al. 2011). While 30 CGG repeats within the 5′ untranslated region of *FMR1* is the modal number of repeats in the general population, expansions in the number of CGG repeats result in a spectrum of conditions, including three distinct disorders—fragile X syndrome (FXS), fragile X-associated primary ovarian insufficiency (FXPOI), and fragile X-associated tremor/ataxia syndrome (FXTAS) (Hagerman and Hagerman 2025). FXS is a neurodevelopmental disorder that is the most common inherited cause of intellectual disability and autism, resulting from expansions of > 200 CGG repeats (Brown 2002). FXS is inherited from a mother who carries an *FMR1* premutation (Nolin et al. 1996), which is defined by 55–200 CGG repeats and occurs in approximately 1 in 150–200 females and 1 in 450 males (Maenner et al. 2013; Seltzer, Baker, et al. 2012; Tassone, Iong, et al. 2012). Women with the *FMR1* premutation are at risk for both FXPOI and FXTAS. FXPOI is the most prevalent genetic cause of impaired ovarian functioning and infertility (Hagerman and Hagerman 2013). FXTAS is a late-onset and progressive neurodegenerative disorder, evident in a subset of premutation carriers after age 50 (Chonchaiya et al. 2010; Hall et al. 2016; Tassone, Greco, et al. 2012).

The symptoms of FXTAS include intention tremor, gait ataxia, neuropathy, memory problems, and executive dysfunction. Considerably more research on FXTAS has been conducted on men than women, due to the more severe manifestation and higher reported prevalence of the syndrome in men. The literature reports that FXTAS affects approximately 40% of premutation carrier men vs. 8%–16% of premutation carrier women over age 50. The lower prevalence and milder severity of FXTAS in women is thought to be at least in part due to the effect of the non-mutated *FMR1* gene on the second X chromosome (Jacquemont, Hagerman, et al. 2004), although additional research on both prevalence and severity in women is needed. After age 50, the diagnosis of FXTAS becomes increasingly likely (Leehey et al. 2008), and is more frequent in those with more than 100 CGG repeats (Tassone et al. 2007). However, some signs and symptoms of FXTAS may appear before age 50, warranting a developmental approach to understanding its emergence during the decades of adulthood. Here we refer to such symptoms as *FXTAS-type symptoms*.

It has been recognized for over 20 years that variability in the phenotypes of women with the premutation may be due to genetic factors other than CGG repeat length as well as environmental factors, and that together these background genetic and environmental factors may moderate *FMR1*-related risk (Jacquemont, Farzin, et al. 2004). Regarding genetic factors, premutation carriers have elevated levels of mRNA, which may be toxic (Berry-Kravis and Hall 2011; Hoem et al. 2011). Additionally, they make longer polyglutamine and other RAN translation products based on the impact of the long CGG repeat in the RNA at the ribosome, which are also potentially toxic (Zhang et al. 2022). Regarding environmental factors that might mitigate symptoms experienced by premutation women, Sodhi and Hagerman (Sodhi and Hagerman 2021) suggested that avoiding environmental toxins (such as general anesthesia) and making lifestyle modifications (such as reducing alcohol consumption and increasing exercise) may reduce the symptoms associated with the premutation. Recent studies have implicated higher education as an additional environmental factor that may reduce the risk of premutation-related symptoms including FXTAS-type symptoms and premature mortality (Hong et al. 2022, 2023), consistent with research on higher education effects on other neurodegenerative diseases (e.g., Cook and Fletcher 2015).

### 1.1 | Present Study

The present longitudinal observational study brings together the genetic risk factor of a family history of FXTAS and the environmental protective factor of higher education to investigate how symptoms related to FXTAS might emerge and increase during midlife and older age in premutation carrier women. Only a few previous studies examined these factors separately; however, in the present study we examine how the interaction between these factors is associated with increased risk for FXTAS-type symptoms in *FMR1* premutation carriers.

To the best of our knowledge, only two previous studies of premutation carrier women have investigated the effects of a family history of FXTAS (Chonchaiya et al. 2010; Klusek et al. 2022), both showing intergenerational risk. Specifically, Chonchaiya et al. (2010) compared premutation carrier daughters of fathers with and without FXTAS and found a significant increase in balance and memory problems in daughters of fathers with FXTAS. Klusek et al. (2022) reported faster age-related decline in syntactic complexity in those with a family history of FXTAS than those without such a family history, starting around age 50. Two other studies investigated higher education effects in carriers of the premutation, showing protective effects. Specifically, Hong et al. (2022). reported significantly more severe FXTAS symptoms in premutation carrier women who did not attain a college degree as compared with those who were college graduates, although the two groups were similar in age, CGG repeats, household income, health behaviors, and general health problems. In another study by Hong et al. (2023). using a different source of data, higher education reduced the risk of early death among adult premutation carriers. This association remained significant even after controlling for IQ and family socioeconomic status measured during high school, as well as factors measured during adulthood (family, psychosocial, health, and financial factors).

Building on these past studies, in the present investigation we investigated factors associated with age-related changes in premutation carrier mothers’ self-reported FXTAS-type symptoms and working memory, measured prospectively over a decade. Using an accelerated longitudinal design approach, we estimated trajectories of change extending from early midlife (age 39) into older age (up to age 80) in a community-based cohort of premutation carrier mothers of adolescents and adults with FXS. We also probed the impact on these trajectories of a family history of FXTAS and college degree attainment as vulnerability and protective factors, respectively.

We conceptualized a family history of FXTAS as a genetic vulnerability factor, and hypothesized that premutation carrier women who had a family history of FXTAS would themselves experience earlier age-related manifestation of FXTAS-type symptoms and limitations in working memory than those who did not have such a family history. As we note below (see Section 4), a family history of FXTAS emerges in tandem with the aging of the premutation woman herself. In this study some women who did not have any relatives with FXTAS at the beginning of the study had relatives who were subsequently diagnosed or became symptomatic during the course of the study. In our conceptualization, the underlying genetic vulnerability existed even though in some cases it preceded knowledge of this family history.

We considered attaining a bachelor’s degree as a protective factor and hypothesized that women who attained a bachelor’s degree (or higher level of education) would experience slower age-related development of FXTAS-type symptoms and working memory difficulties than those who had less than a bachelor’s degree. We focused on bachelor’s degree attainment as distinct from the equivalent years of education without a degree based on past research that found that those who attained a bachelor’s degree had better health (Johnson-Lawrence et al. 2017; Lawrence 2017) and a lower risk of mortality (Krueger et al. 2015) compared with those who did not attain a bachelor’s degree.

Finally, for our key hypothesis, we expected to observe an interaction effect between FXTAS family history and college degree attainment on the age trajectories of the dependent variables. In other words, premutation carriers who had a family history of FXTAS would be more likely to experience elevated FXTAS-type symptoms than those without a family history of FXTAS and these symptoms would emerge at a younger age, unless they had attained a bachelor’s degree.

## 2 | Method

### 2.1 | Participants

Institutional Review Boards from the University of Wisconsin-Madison and the Marshfield Clinic Research Institution approved this study. All participants provided written informed consent prior to initial enrollment in the study and before each wave of data collection.

To be included in the present analysis, participants had to have the *FMR1* premutation and be the biological mother of an adolescent or adult (aged 12+ years) who had the full mutation of FXS. The full mutation status of the adolescent or adult with FXS was verified by medical records. Mothers’ premutation status was verified by medical records and/or molecular assays using buccal samples conducted by the Rush University Medical Center Molecular Diagnostics Laboratory under the direction of Elizabeth Berry-Kravis, MD, PhD. DNA was isolated from buccal samples using standard methods. *FMR1* genotyping to determine CGG repeat length was conducted with the Asuragen AmplideX Kit (Chen et al. 2010; Grasso et al. 2014).

At the beginning of the longitudinal study (Time 1 in 2008), 135 premutation carrier mothers of children with FXS enrolled in the research (Seltzer, Barker, et al. 2012). They were recruited through existing sources—service agencies, clinics, national organizations, and university-based research registries—and lived in 38 US states. For the present analysis, we excluded 13 premutation carrier women whose genetic status was confirmed by medical records but who declined to provide buccal samples and thus had missing data for their specific number of CGG repeats. We also excluded seven women who did not participate in the study after the first point of data collection in this longitudinal study. Thus, the current analysis included data from 115 premutation carrier mothers for whom we had quantification of CGG repeat number and who participated in up to four additional waves of data collection (Times 2–5; 2010–2020). Most (96%) were non-Hispanic Whites, and 62.6% had attained a bachelor’s degree. Their CGG repeats ranged from 67 to 138, with 27.0% having 100 or a greater number of repeats.

Table 1 portrays the number of participants at each wave of data collection between Times 2 and 5, along with average age and age ranges of the mothers at each wave. These women averaged 52.8 years of age at Time 2 and 61.4 years of age at Time 5. Waves of data collection averaged 31 months apart. Table 1 also portrays the average scores on the dependent variables in the present analysis at each of the waves of data collection. Table 2 presents a comparison of women who participated in all four waves of data collection and those with fewer waves of data with respect to key study variables at the start of the study. As shown in Table 2, at the initial point of data collection, mothers who participated in all four waves did not differ from those whose participation ended earlier with respect to FXTAS Family History, educational attainment, age, or CGG repeat length. Additionally, as reported below in Section 3.4, the trajectory analyses restricted to participants who completed all four waves of data collection yielded largely the same pattern of results as those with fewer waves of data.

### 2.2 | Measures

All data used in the present analysis were collected at Times 2 through 5 of the larger study.

#### 2.2.1 | Predictor Variables

The main predictor variable was mothers’ age at each data collection point. Age for each mother was calculated from her date of birth to the date of that mother’s data collection at each wave of the study. Age was then used as a predictor to estimate linear and quadratic terms. Two other key predictors were college degree attainment and a family history of FXTAS

##### 2.2.1.1 | College Degree Attainment.

College degree attainment was coded as 1 = bachelor’s degree or greater educational attainment; 0 = less than a bachelor’s degree.

##### 2.2.1.2 | Family History of FXTAS.

Family history of FXTAS was reported by mothers at each wave of data collection. They were asked if any of their biological relatives (grandparents, parents, or siblings) either had been diagnosed with or were symptomatic for FXTAS (e.g., tremor, ataxia, memory difficulties, etc.). FXTAS family history was coded as a binary variable, with 1 = FXTAS Family History Positive and 0 = FXTAS Family History Negative, reflecting the cumulative data provided by mothers between Times 2 and 5. Both diagnosed and symptomatic FXTAS in relatives were captured as a Positive FXTAS Family History, given that those whose relatives were formally diagnosed with FXTAS and those whose relatives were symptomatic for FXTAS did not differ significantly on any study variable (ps ranged from 0.13 to 0.76).

##### 2.2.1.3 | Control Variables.

Control variables included the mother’s CGG repeat number and CGG repeat squared, as prior studies of women with the premutation revealed non-linear associations for several phenotypes (Dembo et al. 2023; Klusek et al. 2020; Mailick et al. 2014, 2017; Wheeler et al. 2017).

#### 2.2.2 | Dependent Variables

Starting at Time 2, and at each subsequent point of data collection, mothers rated their own FXTAS-type symptoms on items derived from the Chonchaiya et al. (2010) study of premutation symptoms in adult daughters of men with FXTAS. Of the self-report items collected in the Chonchaiya et al. study (Chonchaiya et al. 2010), we included the 14 items that pertained to FXTAS-type symptoms. The 14 self-report items covered four symptom domains (Tremor, Balance, Neuropathy, and Memory), with questions such as “Do you have tremor (shakiness) of the hands?”, “Have you had any problems with your balance?”, “Any problems with burning or pain in the legs?”, “Do you have problems with memory?”, and “Do you get confused or disoriented easily?”. Each item was rated on a scale of 0–2 (0 = no problems, 1 = have problems but not clinically diagnosed or treated with medication for this symptom, and 2 = have clinically diagnosed problems or treated with medication for this symptom) and summed into a total score. Higher total scores signify greater levels of self-reported FXTAS-type symptoms. We analyzed the total score on this measure as well as the scores on each of the four symptom domains.

We also administered the Behavior Rating Inventory of Executive Function-Adult version (BRIEF-A), a reliable and well-validated measure of executive functioning (Roth et al. 2005, 2013). The BRIEF-A is the most commonly used cognitive questionnaire in clinical practice (Rabin et al. 2016). It has been shown to correlate significantly with direct-assessment measures of executive function (e.g., go/no go and trail making tests) in healthy adults (Erkkilä et al. 2018), indicating that the BRIEF-A is an ecologically valid measure of executive function. The BRIEF-A also has been used in past research on women with the premutation (Chan et al. 2017; Maltman et al. 2023). Mothers were asked to indicate the extent to which they experienced each item in daily life on a scale from 1 (never) to 3 (often) in the past month. Scores were converted to *T*-scores (mean = 50, SD = 10), with higher scores indicating more significant deficits in executive functioning.

For the present study, we specifically focused on the Working Memory domain of the BRIEF-A because of its overlap with the memory domain in the measure of FXTAS-type symptoms. The Working Memory domain of the BRIEF-A included eight items that measured difficulties with following directions, concentrating on tasks, especially those that have more than one step, staying on topic, and remembering things even for a few minutes. Notably, unlike the memory items included in the Chonchaiya et al. measure that tapped overall memory difficulties, confusion, and disorientation as perceived by the participant (Chonchaiya et al. 2010) to determine if she was at risk for FXTAS, the Working Memory items in the BRIEF-A specifically reflect the ability *to hold information in mind for further processing, encoding, or mental manipulation*, as measured by items shown to correlate with direct assessment. Thus, the two indicators of memory presented the opportunity to evaluate related but distinct domains that differed in content as well as in method.

### 2.3 | Plan for Analysis

We used an accelerated longitudinal design (ALD; also referred to as a cohort-sequential design or cross-sequential design) to estimate trajectories of FXTAS-type symptoms and limitations in working memory among premutation carrier mothers of individuals with FXS. The ALD estimates a single long-term longitudinal trajectory by combining multiple short-term longitudinal trajectories of each individual covering different ages. This way, the ALD makes it possible to estimate a growth trajectory spanning a wider age range than the duration of time covered by a longitudinal study (Galbraith et al. 2017). As the present analysis covered 10 years and included individuals as young as 39 at Time 2 and as old as 80 at Time 5, using an accelerated longitudinal design enabled estimation of age-related trajectories spanning approximately 40 years.

A series of mixed-effects growth curve models were estimated using chronological age as the time variable. For each outcome, the model selection process began by testing the significance of the random intercept variance and the random slope variance of age to identify the best-fitting model. Subsequently, growth curve models were estimated, incorporating CGG repeat number and the quadratic term of CGG repeats as control variables, linear and quadratic age terms, and interactions between the linear and quadratic age variables, family history of FXTAS, and college degree attainment. These interaction terms were included to examine whether the age-related trajectories of the outcomes differed by family history of FXTAS and college degree attainment. For each dependent variable, results are reported from the growth curve model that included the significant interactions between age, FXTAS family history and college degree attainment. Note, however, preliminary analyses indicated that there were no curvilinear age effects, and therefore the age squared term was dropped from the final analyses. Additionally, in other preliminary analyses, we included the CGG number from the unmutated *FMR1* allele, but the results did not change, so this term was also dropped from the final analysis. The trajectories were estimated for each dependent variable separately in order to evaluate their potentially unique age-related functions. Our goal was to determine for each variable whether there were age-related changes and whether the trajectories differed based on college degree attainment and a family history of FXTAS. Age was centered at 39, and CGG repeat number was centered at the sample mean.

We included all individuals in the study sample in the analysis, regardless of the number of dependent variable data points they contributed, resulting in 115 individuals with 413 data points. Those with just Time 2 data contributed to the estimation of the intercepts and the effect of age at this time point on the intercept, but they did not contribute to estimates of the trajectories. With Maximum Likelihood (ML) estimation, the greater the number of data points, the more influential the case was to the estimation of the age-related trajectories. By fitting our models in a multilevel framework (as opposed to repeated measures ANOVA, for example), we were able to retain all observed data and accommodate missing observations under the same missing at random (MAR) assumptions as are adopted when using full information maximum likelihood (FIML) estimation in other modeling contexts. We set the level of significance at *p* < 0.05. In addition to reporting the unstandardized coefficients in the multilevel models, we also report standardized coefficients to provide an indicator of the practical magnitude in addition to the statistical significance of the effects. Note that although continuous predictors and dependent variables were standardized, binary predictors were unstandardized to facilitate interpretation and reduce multicollinearity.

We present figures that illustrate the results of the statistically significant three-way interactions (College Degree Attainment × FXTAS Family History × Age), consistent with our key hypothesis. Additionally, we estimate the age of participants when the effects of FXTAS Family History became significant. We also conducted two sensitivity analyses, one to check for possible cohort effects in the significant age-related trajectories, and the other to restrict the analyses to the premutation carriers who participated in all four data collection points (*n* = 91) in order to determine if the same pattern of results was obtained as we observed with the full sample of 115 participants. Finally, we conducted a case review of the FXTAS diagnostic status of study participants to determine if they reflected the trends that emerged from the statistical analyses.

Analyses were conducted in Stata 19 (StataCorp 2025). All outcomes were treated as continuous variables.

## 3 | Results

### 3.1 | Change in FXTAS Family History (Time 2 Through Time 5)

At the beginning of the analytic period, 71 participants were classified as FXTAS Family History Negative and 44 were classified as FXTAS Family History Positive. Notably, over the course of the study, 19 of the 71 participants (26.8%) who at Time 1 were classified as FXTAS Family History Negative were reclassified as FXTAS Family History Positive because they had relatives who either were subsequently diagnosed with or became symptomatic for FXTAS during the study period, ultimately resulting in 52 participants who were classified as FXTAS Family History Negative (of whom 20 did not have a college degree and 32 had a college degree) and 63 who were classified as FXTAS Family History Positive (of whom 23 did not have a college degree and 40 had a college degree) (see Table 3).

Of the 63 participants who were FXTAS Family History Positive, the premutation was inherited from the mother’s side of the family in 25 cases, while in 36 cases it was inherited from the father’s side of the family (in two cases the inheritance could not be determined).

### 3.2 | Trajectories of Age-Related Change

#### 3.2.1 | Self-Reported FXTAS-Type Symptoms

Table 4 reports the results of the ALD analyses for mothers’ own FXTAS-type symptoms as measured by the Chonchaiya et al. (2010) study measure and the Working Memory measure included in the BRIEF-A. Regarding the Total score of the measure of FXTAS-type symptoms, there was a significant three-way interaction between FXTAS Family History, College Degree Attainment, and Age, consistent with our key hypothesis (see Table 4, Column A). As illustrated in Figure 1, the two groups of mothers who were FXTAS Family History Positive had age-related trajectories of significantly worsening FXTAS-type symptoms, but these two groups diverged depending on whether the mothers had attained a college degree. Those without a college degree had steep age-related increases in their Total FXTAS-type symptoms, while such symptoms in those who had attained a college degree increased more gradually with advancing age. Thus, college degree attainment moderated the genetic risk of a family history of FXTAS. In contrast, the age-r elated trajectories of the two groups who were FXTAS Family History Negative were not significant, remaining relatively low across the age range, regardless of college degree attainment. The magnitude of the standardized three-way interaction (*β* = −0.59) suggested a medium effect size in age-related change in FXTAS-type symptoms across different combinations of FXTAS Family History and College Degree Attainment.

Similar patterns were observed for the Tremor and Memory symptom domains of the Chonchaiya et al. study’s (2010) measure (see Table 4, Column B and E, respectively), where there were significant three-way interactions between FXTAS Family History, College Degree Attainment, and Age (see Figures 2 and 3). For these symptom domains, it was only the premutation carrier mothers who were FXTAS Family History Positive and who did not attain a college degree for whom there was a significant worsening of symptoms with advancing age. The other mothers in the sample had low levels of tremors and memory difficulties that were relatively constant across the age range. The standardized three-way interaction coefficients for the Tremor and Memory symptom domains (*β* = −0.71 and *β* = −0.52, respectively) suggested medium effect sizes in age-related change in these outcomes across levels of FXTAS Family History and College Degree Attainment.

For the symptom domain of Balance (Table 4, Column C), there were separate significant two-way interaction effects of College Degree Attainment × Age and FXTAS Family History × Age, but the three-way interaction effect of college degree attainment on balance symptoms was not observed. For the symptom domain of Neuropathy (Table 4, Column D), only the two-way interaction effect of College Degree Attainment × Age was significant. For these two-way interactions, those who were FXTAS Family History Positive had significantly increasing symptoms with advancing age, as did those who did not have a college degree. Notably, the standardized coefficients associated with these two-way interactions were substantially lower than those reported above for the three-way interactions, indicating small effect sizes in age-related change in these variables across levels of FXTAS Family History and College Degree Attainment.

#### 3.2.2 | Working Memory

Table 4, Column F reports the results of the ALD analysis for mothers’ Working Memory limitations derived from the BRIEF-A. Counter to our hypothesis, the three-way interaction was not significant, although there were significant two-way interactions (College Degree Attainment × Age; FXTAS Family History × Age). For these two-way interactions, those who were FXTAS Family History Positive had significantly worsening symptoms with advancing age, as did those who did not have a college degree. However, the standardized coefficients associated with these two-way interactions were substantially lower than those reported above for the three-way interactions, suggesting a small effect size in age-related change in working memory limitations across levels of FXTAS Family History and College Degree Attainment.

### 3.3 | Probing the Regions of Significance of FXTAS Family History

For each of the significant three-way interaction effects, we estimated the marginal effects of college degree attainment and age on the outcome variables separately for mothers with FXTAS Family History Positive and Negative. Following the approach used by Preacher et al. (2006) to identify the ranges of age where the differences in the outcome variables for mothers with and without a college degree become statistically different from zero, plots were constructed that illustrated how the college degree effects (i.e., difference between mothers with a college degree and without a college degree) on outcomes varied by age, separately for mothers who were FXTAS Family History Positive and Negative. We then examined the 95% confidence intervals of these college education effects across the age range.

Regarding the Total score of the measure of self-reported FXTAS-type symptoms, for mothers who were FXTAS Family History Positive, the age trajectory of those without a college degree worsened significantly starting at age 51 as compared with those who had a college degree. However, for those who were FXTAS Family History Negative, the college degree effects were not significant at any time across the age range of the study.

The patterns for the Tremor and Memory domains were similar to the Total scale, where the age trajectory of those who were FXTAS Family History Positive worsened significantly starting at age 52 and age 53, respectively, if they did not have a college degree, as compared with those who had a college degree. However, for those who were FXTAS Family History Negative, the college degree effects were not significant at any age.

Additionally, we probed the regions of significance for the two-way interactions of FXTAS Family History × Age. With respect to the Balance domain of the FXTAS-type symptom measure, even though the two-way interaction was significant, the difference between those who were FXTAS Family History Positive versus those who were FXTAS Family History Negative was not significant at any age. However, with respect to the Working Memory domain of the BRIEF-A, women who were FXTAS Family History Positive worsened significantly after age 76 as compared with women who were FXTAS Family History Negative.

### 3.4 | Sensitivity Analyses

#### 3.4.1 | Cohort Effects Analysis

The goal of this first sensitivity analysis was to determine if there were cohort effects in the significant age-related trajectories (e.g., to ascertain whether mothers who were early in midlife at the start of the study showed a different pattern of change as compared to those who were approaching older age when the study began). For this analysis, we included an interaction term between age at the first point of data collection and the trajectory term for each dependent variable. A significant interaction would suggest differences in trajectories between those who were younger versus older when the study began. However, no cohort effects were observed.

#### 3.4.2 | Complete Cases Analysis

The goal of this second sensitivity analysis was to determine whether restricting the analysis to data collected from only the 91 mothers who participated at all time points (Time 2 through Time 5) would yield the same pattern of results as the full ALD analyses that included all 115 mothers. The results with the 91 mothers largely confirmed the patterns of the ALD analysis that included all 115 cases at either the *p* < 0.05 level of significance or at a trend level (*p*s = 0.053–0.071), see Table S1. It should be noted, however, that the three-way interaction model for the Chonchaiya et al. measure of FXTAS-type memory symptoms did not converge in the complete cases analysis and was replaced by a two-way interaction model.

#### 3.4.3 | Case Review

Following our statistical analyses, we conducted a case review of the FXTAS diagnostic status of study participants. By Time 5, seven of the premutation carriers in the present sample reported that they had recently received a diagnosis of FXTAS, all of whom were age 50 or older (mean = age 69.2) with an average of 98.2 CGG repeats (range = 83–115 repeats). All but one of these women had a family history of FXTAS and all but two did not attain a bachelor’s degree, reflecting the associations of age, family history, and college education observed in the statistical analyses.

## 4 | Discussion

The premutation is inherited intergenerationally and therefore it is not surprising that a family history of FXTAS might increase the likelihood for premutation carrier women to experience their own age-related decline. Indeed, a family history of FXTAS has been shown in previous studies to be a risk factor for elevated balance, memory, menopausal, and linguistic symptoms in women with the premutation (Chonchaiya et al. 2010; Klusek et al. 2022). Separately, attaining a college degree has been identified in previous studies as a possible protective factor for premutation carrier women that appears to reduce the risk of the emergence their own FXTAS-type symptoms and with earlier mortality (Hong et al. 2022, 2023). Notably, previous studies of family history effects have included higher educational attainment as a covariate (e.g., Klusek et al. 2022). However, to the best of our knowledge, the present study is the first to simultaneously investigate whether the risk factor of a family history of FXTAS and the protective factor of college degree attainment *interact* to affect aging-related phenotypes in premutation women. The results reported here regarding the interaction between these two factors advance understanding of the heterogeneity of the premutation phenotype in women.

Using an approach that estimated trends over a 40-year period during midlife and older age, the present research showed that a positive family history of FXTAS was associated with a greater worsening in FXTAS-type symptoms in mothers of children with FXS during their early 50s, and that attaining a college degree was linked with less risk. This protective effect was evident for the Total score and for the Tremor and Memory domains of the Chonchaiya study’s (Chonchaiya et al. 2010) measure of FXTAS-type symptoms. Notably, Klusek et al. (2022) also reported earlier decline in linguistic abilities associated with FXTAS family history for premutation carrier women in their 50s, signaling the onset of risk for these women that may ultimately progress to FXTAS for some. Recently, Liani et al. (2025) reported a case series of 10 women with what they termed “*preFXTAS”*, a set of symptoms in women with the premutation manifested earlier than the typical age when FXTAS is diagnosed, conceptualizing FXTAS-type symptoms more as a continuum of symptoms than as a binary diagnosis, and possibly including different symptoms in women than in men.

The present study is not the only example of a college degree moderating a significant genetic risk. Cook and Fletcher (2015) presented evidence using population-level data that the genetic effect of the *APOE*4 variant on age-related cognitive decline was absent in individuals who completed college (versus high school graduates); in other words, completing college “rescued” the genetic effects of the risk allele. Further, Cook and Fletcher were able to control for possible alternative explanations, showing that the protective influence of a college degree was net of increased income, better access to medical care, job characteristics, and other personal or social factors. They interpreted the results to suggest that attaining the degree enhanced cognitive reserve. Other studies have shown an association between college degree attainment and reduced or delayed symptoms of neurodegenerative diseases, such as Alzheimer’s and dementia (Aiello Bowles et al. 2019; Iraniparast et al. 2022), Parkinson’s disease (Blume et al. 2017), as well as other neurological signs and symptoms (Agrawal 2009). Taken together, these findings suggest that a college education may have broad neuroprotective effects by contributing to cognitive reserve. Nevertheless, it will be important for future research to replicate these findings and to continue to investigate other protective factors for premutation carriers who bear this familial risk. For example, research on the neuroprotective effects of exercise, which has been demonstrated for other neurodegenerative diseases (Johansson et al. 2022; Langeskov-Christensen et al. 2024; Lopez-Ortiz et al. 2023; Sujkowski et al. 2022) should be probed for FXTAS, as has been suggested by Sodhi and Hagerman (2021).

The longitudinal design of the present study revealed the extent to which a family history of FXTAS increases over time. We found that more than one-quarter of women who at the beginning of this decade-long study period did not have a family history of FXTAS had relatives who were subsequently diagnosed with or became symptomatic for this condition during the course of the present research. Separately, some participants who were categorized as FXTAS Family History Negative throughout the study period might actually have had a positive family history, unbeknownst to them. This situation may arise when older family members of study participants (grandparents and parents) were deceased either before 2001 when FXTAS was first reported (Hagerman et al. 2001) or before 2008 when the present research began. Thus, obtaining a formal diagnosis of FXTAS would not have been possible or even likely for them. Also, as both Chonchaiya et al. (2010) and Klusek et al. (2022) noted, some research participants who report no family history of FXTAS will have a family member develop FXTAS after a study ends due to an incomplete observation period. Nevertheless, despite these challenges in identifying a family history of FXTAS, the strong pattern of statistical associations reported here supports the risks of a positive FXTAS family history. Thus, cross-sectional studies that examine a family history of FXTAS at a single point in time can provide only a snapshot of this intergenerational risk that in fact evolves as the generations of a family age.

### 4.1 | Limitations, Strengths, and Future Directions

This study is not without limitations. Importantly, this is an observational study, so causal inference is not supported by this research design. Although the data analytic approach allowed for the inclusion of the entire longitudinal sample even if they did not participate in each study wave, with nearly 80% of the sample retained over the full decade-long study, attrition remains a limitation. Yet the complete cases analysis largely confirmed the results. Nevertheless, replication of the results in future research is warranted.

Additionally, the present study participants may not be representative of the true population of premutation women for several reasons. All had a child with FXS, and therefore they may not be representative of undiagnosed premutation women, or those who did not have children, or those whose children did not have FXS. Caring for a child with FXS has been shown to be stressful for the family, especially if they have behavior problems which are prevalent in FXS (Hall et al. 2007; Wheeler et al. 2014). Thus, child-related stress may compound the effects of a family history of FXTAS. All participants volunteered for the research, and most remained active participants in the study for over a decade. Most (63%) had a college degree, a percentage considerably higher than the US population.

Another limitation is that we did not have a measure of the IQ of the participants, which should be controlled in future research, as IQ might be associated with college degree attainment. However, as Klusek et al. (2026) commented in her study of cognitive dysfunction in premutation carrier women, “socioeconomic advantages tied to higher education (e.g., better medical care, cognitively-stimulating occupations) could also contribute to neuroprotection, rather than the educational experience of college itself.”

Although a sample of 115 participants with over 400 data points is not small in size for studies of this population, future research that includes a larger and more diverse sample is needed. A larger sample size in future research would make it possible to conduct more complex statistical modeling and thereby include interactions of age with child-related stress (such as behavior problems) in addition to FXTAS family history and college degree attainment. Although preliminary analyses of the interactions between CGG-repeats, FXTAS family history, and college degree attainment in the present study were not significant, these might be detectable with a larger sample size, with other measures, and by including activation ratio data. (See Table S2 for the results of the preliminary analysis including interactions with CGG repeats and CGG repeats squared in the age trajectory analyses.) Thus, results warrant replication using larger samples, a broader range of moderators, outcomes, and additional genetic data.

Apart from CGG repeat testing results and medical records obtained for the premutation carrier mother and her son or daughter with FXS, data for this study were self-reported, including having a family history of FXTAS. It is possible that knowledge of a family history of FXTAS could result in differential symptoms reporting for those who were positive versus negative for a family history, which could inflate the family history effect. Although patient report is a common method for obtaining family history information, medical confirmation of family history status in future research would confirm the conclusions. However, there are barriers that members of an extended family might face in obtaining a medical diagnosis of FXTAS, including limited access to specialized clinics, leaving patient report as an important source of information. Additionally, studies extending over longer periods of time would further strengthen the data, as the variables included in the present analysis reflect age-related processes.

Juxtaposed against these limitations were the contributions of the present research. It is one of the few studies to have traced the development of FXTAS-type symptoms in premutation carrier women over a long stretch of midlife and into older age, pointing to the timing of vulnerability and protective factors, and it probed how genetic and environmental factors interact to yield insights about the heterogeneity of the premutation phenotype in women. We employed different approaches to ascertain associations, including statistical analyses and case reviews that largely confirmed the risk and protective factors of a family history of FXTAS and attainment of a college degree. The seven participants who had reported that they had been subsequently diagnosed with FXTAS represented 6.8% of the present sample members age 50 and older. Although this is a somewhat lower percentage than has been reported in past research, their average age was relatively young, so it is possible that some additional sample members will be diagnosed in the future. Further, most of these women who were diagnosed with FXTAS had a positive family history but did not have a college degree, supporting trends observed in the statistical analysis.

There are many unanswered questions that should be addressed in future research. For example, why is college degree attainment protective for some FXTAS-type symptoms but not for all? This was particularly evident with respect to the general memory items in the Chonchaiya study’s (Chonchaiya et al. 2010) measure versus the working memory domain of the BRIEF-A, where college degree attainment was protective for the former but not for the latter. The latter group (those with a positive FXTAS Family History and no college degree) warrant closer study in future research with more robust measures, as in the current study they were particularly vulnerable with respect to working memory (see scatterplot, Figure S1).

An additional unanswered question is at what age it is appropriate to make conclusions about whether a woman with the premutation has a family history of FXTAS? Given that this family history variable emerges in tandem with the aging of the premutation woman herself, is it ever valid to conclude that a woman has no family history of FXTAS without full family cascade testing? A related question warranting examination in future research concerns the phenotypic significance of the inheritance patterns of the family history of FXTAS (maternally vs. paternally inherited), as affected relative type carries different genetic risk implications.

### 4.2 | Conclusion

In conclusion, women with the *FMR1* premutation who have a family history of FXTAS may be at risk for developing similar symptoms in late midlife into older age. Yet this inherited risk may be partially mitigated by completing a college education. These results have implications for the decisions made by adolescents who carry the premutation regarding the value of a college education and more generally suggest that cognitive enrichment during the period of neuroplasticity might provide additional protection from the neurodegenerative symptoms that are experienced by some women with the premutation as they age.

## Supplementary Material

Supplemental Material

Additional supporting information can be found online in the Supporting Information section. **Table S1:** Age trajectory estimates from the growth curve model predicting outcomes: Complete cases analysis (*n* = 91).^1^ **Table S2:** Age trajectory estimates from the growth curve model predicting outcomes: CGG repeat length interaction analysis.^1^ **Figure S1:** Scatterplot and lowess fitted line of BRIEF-a working memory scores of premutation carrier women with positive family history of FXTAS and no college degree.

## Figures and Tables

**FIGURE 1 |:**
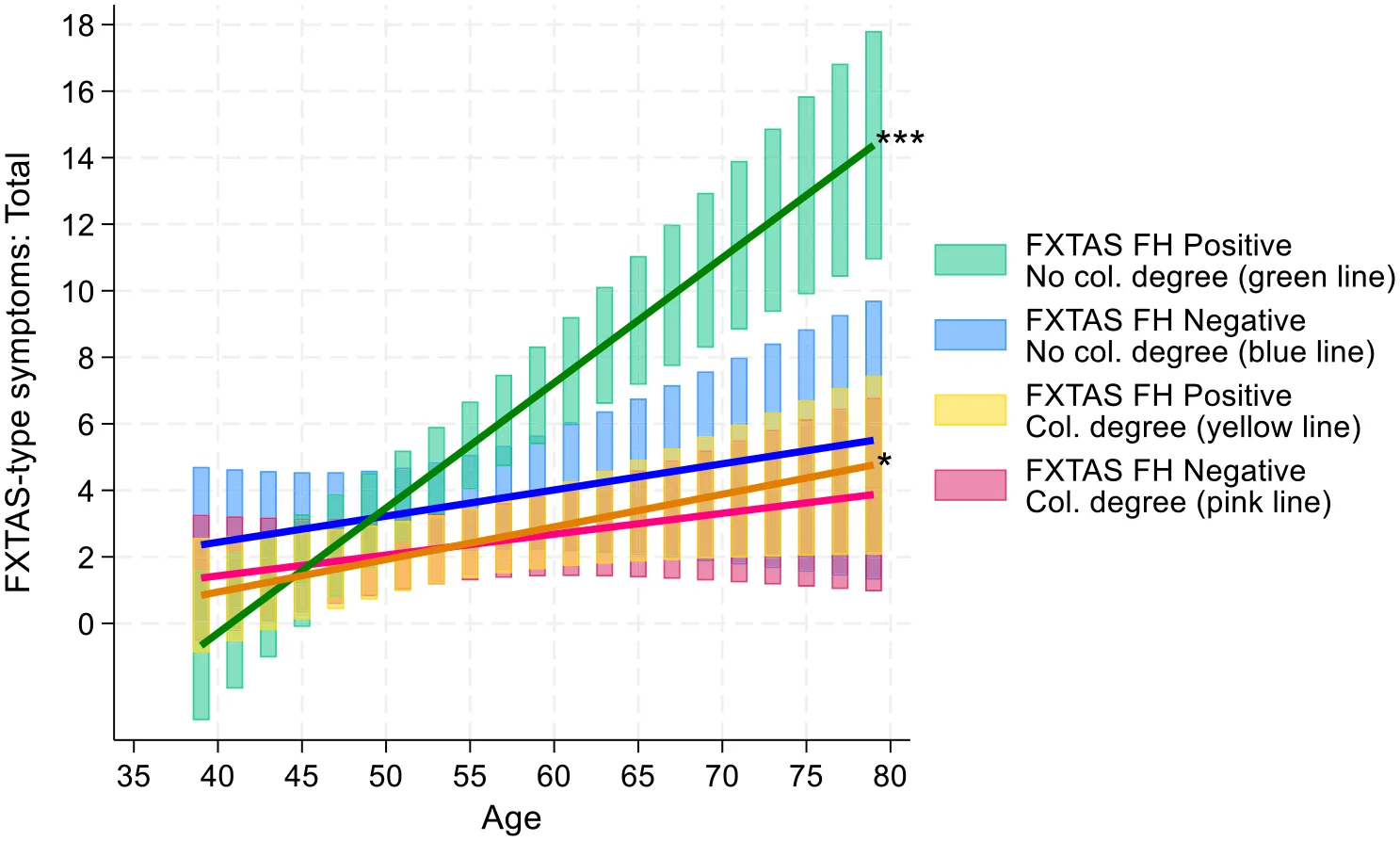
Age trajectory of FXTAS-type symptoms total score with 95% confidence intervals, by college degree attainment and FXTAS Family History. Significance of simple slope * *p* < 0.05, *** *p* < 0.001. FH, Family History.

**FIGURE 2 |:**
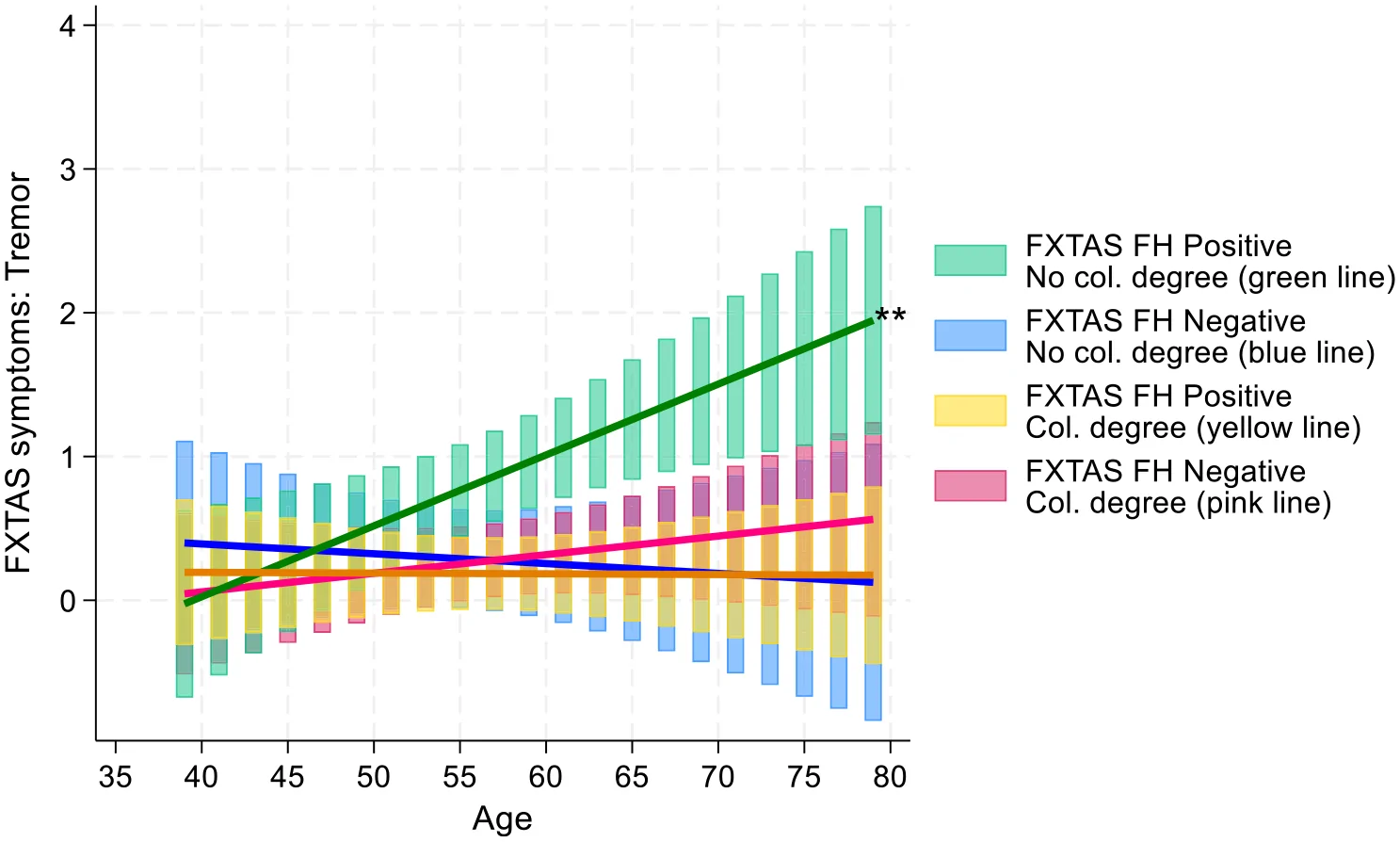
Age trajectory of FXTAS-type symptoms tremor domain with 95% confidence intervals, by college degree attainment and FXTAS Family History. Significance of simple slope ** *p* < 0.01. FH, Family History.

**FIGURE 3 |:**
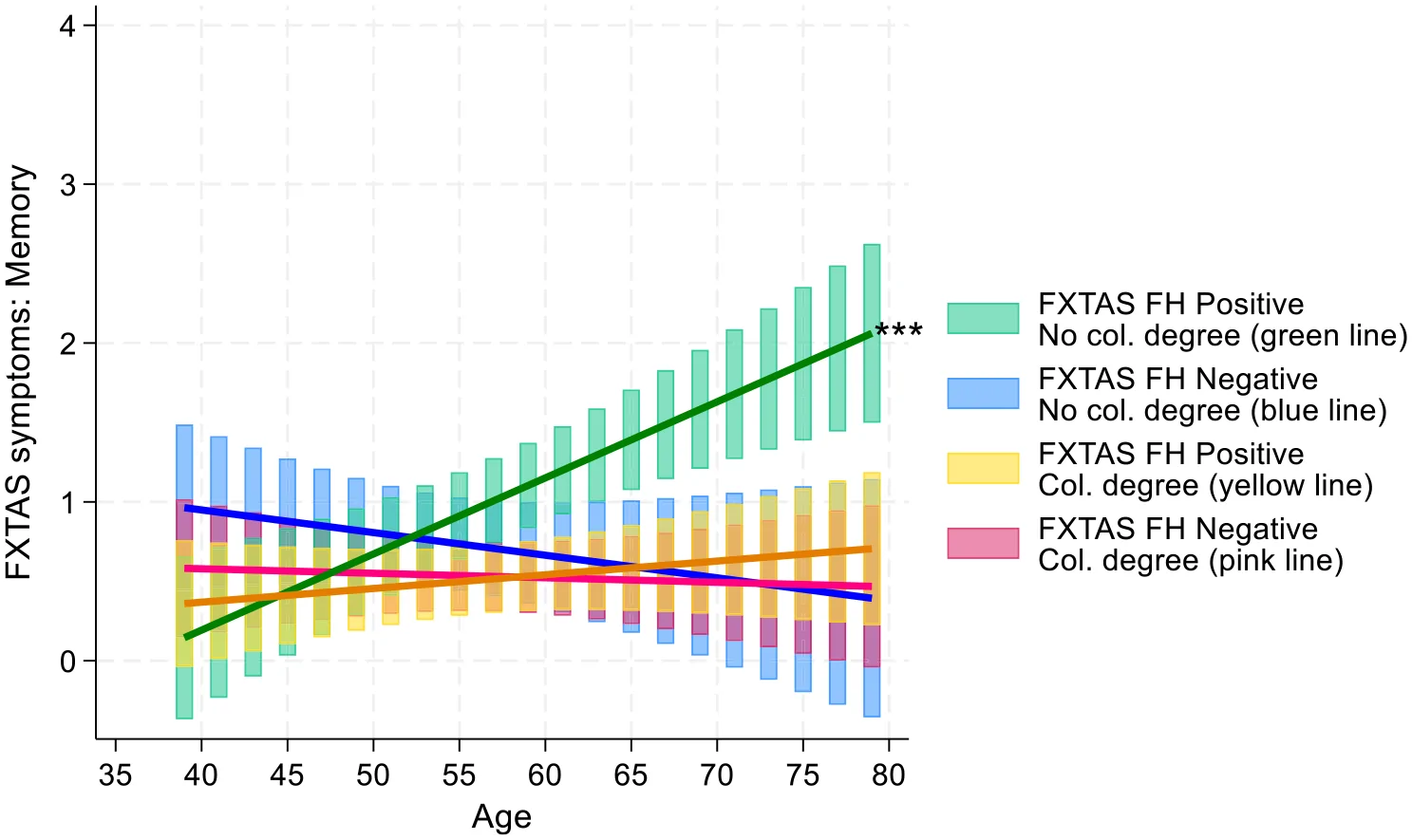
Age trajectory of FXTAS-type symptoms memory domain with 95% confidence intervals, by college degree attainment and FXTAS Family History. Significance of simple slope *** *p* < 0.001. FH, Family History.

**TABLE 1 |:** Data collection periods, and descriptive statistics of study variables by study waves.^a^

	Waves of data collection (*n*)			
	Time 2 (115)	Time 3 (107)	Time 4 (100)	Time 5 (91)
Dates of data collection	November 2010–November 2011	July 2012–April 2013	August 2016–April 2017	September 2019–May 2020
Study variables				
Age of premutation carrier women	52.8 (7.3) [39.0, 72.3]	54.6 (7.3) [40.4, 73.7]	58.4 (7.2) [44.2, 76.6]	61.4 (7.1) [47.3, 79.7]
FXTAS-type symptoms				
Total	2.53 (3.1) [0, 12]	2.47 (3.1) [0, 19]	2.76 (3.0) [0, 14]	3.96 (4.6) [0, 21]
Tremor	0.27 (0.8) [0, 5]	0.22 (0.7) [0, 5]	0.26 (0.8) [0, 5]	0.34 (0.9) [0, 5]
Balance	0.84 (1.5) [0, 6]	0.83 (1.5) [0, 8]	1.04 (1.4) [0, 6]	1.66 (2.2) [0, 10]
Neuropathy	0.85 (1.3) [0, 6]	0.79 (1.3) [0, 6]	0.91 (1.2) [0, 5]	1.26 (1.7) [0, 6]
Memory	0.58 (0.8) [0, 4]	0.62 (0.7) [0, 3]	0.55 (0.6) [0, 2]	0.69 (0.9) [0, 4]
Executive functioning limitations				
Working memory	57.4 (14.1) [39, 97]	57.2 (14.7) [40, 96]	56.8 (12.9) [40, 89]	58.4 (13.2) [40, 95]

a For the study variables, means, standard deviations (in parentheses), and ranges (in brackets) are presented.

**TABLE 2 |:** Comparisons of study variables at the baseline (Time 2) by study participation status: complete cases vs. attrition.^a^

	Complete cases (*n* = 91)	Attrition cases (*n* = 24)	*t*-value/Chi-square
Maternal age	52.5 (7.1) [39, 71]	52.2 (8.2) [40, 72]	*t* = 0.169
% College degree	64.8%	54.2%	*χ*^2^ = 0.337
% positive family history of FXTAS	58.2%	41.7%	*χ*^2^ = 0.147
CGG repeat length	94.9 (16.3) [67, 138]	92.2 (15.5) [68, 135]	*t* = −0.717

a Means, standard deviations (in parentheses), and ranges (in brackets) are presented.

**TABLE 3 |:** Participants cross-classification by college degree attainment and FXTAS Family History.

	FXTAS Family History Negative	FXTAS Family History Positive	Total
No college degree	20 (17.4%)	23 (20.0%)	43 (37.4%)
College degree	32 (27.8%)	40 (34.8%)	72 (62.6%)
Total	52 (45.2%)	63 (54.8%)	115 (100.0%)

**TABLE 4 |:** Age trajectory estimates from the growth curve model predicting outcomes.^a^

	FXTAS-type symptoms (Chonchaiya et al. 2010)					BRIEF-A
	A. Total score	B. Tremor	C. Balance	D. Neuropathy	E. Memory	F. Working memory
CGG repeats	0.02 (0.02) [0.07]^b^	0.01 (0.01) [0.18]	0.02 (0.01) [0.14]	−0.01 (0.01) [−0.06]	0.00 (0.00) [0.06]	−0.09 (0.09) [−0.10]
CGG repeats-squared	−0.00 (0.00) [−0.06]	−0.00 (0.00) [−0.07]	−0.00 (0.00) [−0.06]	−0.00 (0.00) [−0.02]	−0.00 (0.00) [−0.04]	0.00 (0.00) [−0.05]
College degree (yes = 1)	−1.00 (1.53) [−0.36]	−0.35 (0.46) [−0.02]	0.07 (0.38) [−0.49]	0.35 (0.37) [−0.59]	−0.38 (0.35) [−0.24]	5.87 (3.71) [−0.21]
FXTAS Family History (yes = 1)	−3.03 (1.61) [0.58]	−0.42 (0.48) [0.69]	−0.78 (0.39)* [0.10]	0.29 (0.21) [0.21]	−0.82 (0.37)* [0.31]	−5.85 (3.69) [0.01]
Age	0.08 (0.07) [0.18]	−0.01 (0.02) [−0.07]	0.07 (0.03)** [0.34]	0.08 (0.02)*** [0.47]	−0.01 (0.01) [−0.15]	0.24 (0.17) [0.14]
Age × College degree	−0.02 (0.09) [−0.04]	0.02 (0.02) [0.20]	−0.05 (0.03)* [−0.25]	−0.07 (0.02)** [−0.39]	0.01 (0.02) [0.12]	−0.51 (0.17)** [−0.29]
Age × FXTAS Family History	0.30 (0.10)** [0.67]	0.06 (0.03)* [0.57]	0.06 (0.03)* [0.27]	—	0.06 (0.02)** [0.64]	0.35 (0.17)* [0.20]
College degree × FXTAS Family History^c^	2.51 (2.05) [−0.56]	0.57 (0.61) [−0.79]	—	—	0.60 (0.46) [−0.34]	—
Age × FXTAS Family History × College degree	−0.26 (0.12)* [−0.59]	−0.07 (0.03)* [−0.71]	—	—	−0.05 (0.02)* [−0.52]	—
Constant	2.35 (1.19)* [0.23]	0.39 (0.36) [0.01]	0.50 (0.36) [0.39]	0.02 (0.32) [0.35]	0.96 (0.27)*** [0.15]	54.5 (3.56)*** [0.09]

*Note*: Unstandardized coefficients are presented with robust standard errors in parentheses. Standardized coefficients are presented in brackets.

*** *p* < u.uui.

** *p* < 0.01.

* *p* < 0.05.

a For all FXTAS-type symptom scores, the random coefficient models were estimated. For the BRIEF-A Working memory scale, the random intercept model was estimated. Age was centered at 39, the youngest age at Time 2, and CGG repeat number was centered at the sample mean.

b For the calculation of standardized coefficients, continuous predictors and dependent variables were standardized, whereas binary predictors were left unstandardized to facilitate interpretation and reduce multicollinearity.

c Sample size in each group of FXTAS family history and college degree cross-classification: FXTAS Family history negative with no college degree (*n* = 20)/FXTAS Family history negative with a college degree (*n* = 32). FXTAS Family history positive with no college degree (*n* = 23)/FXTAS Family history positive with a college degree (*n* = 40).

## Data Availability

The dataset analyzed in this study is not publicly available per IRB. Due to the sensitive nature of the study, participants were assured raw data would not be shared.
