# Supplementary material for: Interaction of FXTAS Family History and College Degree Attainment Predicts Trajectories of Cognitive and Motor Symptoms in FMR1 Premutation Carrier Women
Source: Am J Med Genet B Neuropsychiatr Genet. Author manuscript; Available in PMC 2026 Oct 12. (PMC13656318; doi:10.1002/ajmg.b.70022)
Supplement: Supplementary file 1 [file NIHMS2214149-supplement-Supplemental_Material.docx]

**Supplemental Material**

Table S1. Age trajectory estimates from the growth curve model predicting outcomes: Complete cases analysis (n = 91)^1^

|  | FXTAS-type Symptoms (Chonchaiya et al., 2010) | | | | | BRIEF-A |
| --- | --- | --- | --- | --- | --- | --- |
|  | A.  Total score | B. Tremor | C. Balance | D. Neuropathy | E. Memory | F. Working memory |
| CGG repeats | 0.03 (0.02) | 0.01 (0.01) | 0.02 (0.01) | 0.00 (0.01) | 0.00 (0.00) | -0.05 (0.09) |
| CGG repeats-squared | -0.00 (0.00) | -0.00 (0.00) | -0.00 (0.00) | -0.00 (0.00) | -0.00 (0.00) | -0.00 (0.00) |
| College degree (yes = 1) | -0.95 (1.05) | -0.22 (0.51) | 0.06 (0.41) | 0.14 (0.31) | -0.13 (0.22) | 1.29 (3.88) |
| FXTAS Family History (yes = 1) | -2.52 (1.17) | -0.36 (0.53) | -0.50 (0.42) | 0.28 (0.19) | -0.53 (0.23)* | -5.22 (3.87) |
| Age | 0.09 (0.07) | -0.00 (0.02) | 0.07 (0.03)** | 0.09 (0.02)*** | 0.01 (0.01) | 0.25 (0.17) |
|  |  |  |  |  |  |  |
| Age x  College degree | -0.02 (0.09) | 0.02 (0.03) | **-0.05 (0.03)+** | **-0.07 (0.02)**** | 0.01 (0.01) | **-0.43 (0.17)*** |
| Age x  FXTAS Family History | 0.29 (0.09)** | 0.06 (0.03)* | **0.05 (0.03)+** | -- | **0.03 (0.01)**** | **0.32 (0.17)+** |
| College degree x  FXTAS Family History | 2.22 (1.44) | 0.57 (0.66) | -- | -- | -- | -- |
|  |  |  |  |  |  |  |
| Age x  FXTAS Family History x  College degree | **-0.25 (0.11)*** | **-0.07 (0.03)*** | -- | -- | -- | -- |
|  |  |  |  |  |  |  |
| Constant | 1.63 (0.83)* | 0.09 (0.42) | 0.28 (0.39) | 0.04 (0.27) | 0.73 (0.22)** | 56.4 (3.87)*** |

Notes. Regression coefficients are presented with robust standard errors in parentheses.

*** p < 0.001, ** p < 0.01, * p < 0.05, + p < 0.10.

1. For all FXTAS-type symptom scores, the random coefficient models were estimated. For the BRIEF-A working memory scale, the random intercept model was estimated. Age was centered at 39, the youngest age at the baseline.

2. For FXTAS-type memory symptoms score, the three-way interaction model did not converge. Here, therefore, results from two two-way interactions model result was reported.

Table S2. Age trajectory estimates from the growth curve model predicting outcomes: CGG repeat length interaction analysis^1^

|  | FXTAS-type Symptoms (Chonchaiya et al., 2010) | | | | | BRIEF-A |
| --- | --- | --- | --- | --- | --- | --- |
|  | A. Total score | B. Tremor | C. Balance | D. Neuropathy | E. Memory | F. Working memory |
| CGG repeats | -0.01 (0.07) | -0.00 (0.02) | 0.01 (0.03) | -0.01 (0.03) | -0.03 (0.02) | -0.04 (0.26) |
| CGG repeats^2 | -0.00 (0.00) | -0.00 (0.00) | -0.00 (0.00) | -0.00 (0.00) | -0.00 (0.00) | -0.01 (0.01) |
| College degree (yes = 1) | -0.23 (1.27) | -0.18 (0.39) | -0.09 (0.48) | 0.27 (0.44) | -0.29 (0.28) | -4.43 (4.61) |
| FXTAS FH (yes = 1) | -2.65 (1.30)* | -0.22 (0.40) | -0.95 (0.51) | -0.69 (0.46) | -0.64 (0.29)* | -3.72 (4.72) |
| Age | 0.16 (0.08)* | 0.01 (0.02) | 0.08 (0.03)* | 0.04 (0.03) | 0.01 (0.01) | 0.04 (0.22) |
| Age x College degree | -0.16 (0.08)* | -0.01 (0.02) | -0.06 (0.03) | -0.06 (0.03)* | -0.02 (0.01) | -0.23 (0.22) |
| Age x FXTAS FH | 0.20 (0.08)* | 0.02 (0.02) | 0.06 (0.03) | 0.07 (0.03)* | 0.04 (0.01)** | 0.40 (0.22) |
| Age x CGG repeats | 0.00 (0.00) | 0.00 (0.00) | 0.00 (0.00) | 0.00 (0.00) | 0.00 (0.00)** | 0.01 (0.01) |
| Age x CGG repeats^2 | 0.00 (0.00) | 0.00 (0.00) | -0.00 (0.00) | 0.00 (0.00) | -0.00 (0.00) | 0.00 (0.00) |
| College degree x  CGG repeats | -0.03 (0.08) | -0.02 (0.02) | -0.03 (0.04) | -0.01 (0.03) | 0.02 (0.02) | -0.48 (0.30) |
| College degree x  CGG repeats^2 | 0.00 (0.00) | 0.00 (0.00) | 0.00 (0.00) | 0.00 (0.00) | 0.00 (0.00) | **0.04 (0.01)***** |
| Age x College degree x  CGG repeats | -0.00 (0.01) | 0.00 (0.00) | 0.00 (0.00) | 0.00 (0.00) | **-0.00 (0.00)*** | 0.01 (0.01) |
| Age x College degree x  CGG repeats^2 | -0.00 (0.00) | -0.00 (0.00) | 0.00 (0.00) | -0.00 (0.00) | 0.00 (0.00) | **-0.00 (0.00)*** |
| FXTAS FH x  CGG repeats | 0.00 (0.08) | 0.02 (0.02) | 0.01 (0.03) | -0.02 (0.03) | 0.01 (0.02) | 0.30 (0.29) |
| FXTAS FH x  CGG repeats^2 | 0.01 (0.00) | 0.00 (0.00) | 0.00 (0.00) | 0.00 (0.00) | 0.00 (0.00) | -0.01 (0.02) |
| Age x FXTAS FH x  CGG repeats | -0.00 (0.00) | -0.00 (0.00) | -0.00 (0.00) | 0.00 (0.00) | -0.00 (0.00) | -0.01 (0.01) |
| Age x FXTAS FH x  CGG repeats^2 | -0.00 (0.00) | -0.00 (0.00) | -0.00 (0.00) | -0.00 (0.00) | -0.00 (0.00) | -0.00 (0.00) |
| Constant | 2.15 (1.20) | 0.43 (0.38) | 0.56 (0.43) | 0.56 (0.40) | 0.84 (0.27)* | 58.96 (4.31)* |

Notes. Regression coefficients are presented with robust standard errors in parentheses.

*** p < 0.001, ** p < 0.01, * p < 0.05. FH: Family History.

1. For all FXTAS-type symptom scores, the random coefficient models were estimated. For the BRIEF-A working memory scale, the random intercept model was estimated. Age was centered at 39, the youngest age at the baseline.

Figure S1. Scatterplot and Lowess Fitted Line of BRIEF-A Working Memory Scores of Premutation Carrier Women with Positive Family History of FXTAS and No College Degree.
